# Public safety messaging during super bowl LVI: A pilot study and modified framework

**DOI:** 10.1371/journal.pone.0284921

**Published:** 2023-04-25

**Authors:** Janine Cadet, Soraya Sutherlin, Rita V. Burke

**Affiliations:** 1 Keck School of Medicine USC, Los Angeles, CA, United States of America; 2 Emergency Management Safety Partners, Los Angeles County, CA, United States of America; University of Malta, MALTA

## Abstract

**Objective:**

The Super Bowl is one of the most widely attended sporting events and requires proper communication to ensure emergency preparedness for everyone in the city. This pilot study used Super Bowl LVI as a setting to inform future research evaluating the effectiveness of the public health messaging during mass-gathering events.

**Methods:**

This pilot study modifies past theoretical frameworks and research tools to create a novel survey instrument focused on public safety message effectiveness. This survey was sent to all of those that opted-in to the Joint Information Center’s notification platform during Super Bowl LVI.

**Results:**

The results show that message comprehension, source credibility, and perceived risk might not be associated with proactive behavior for public safety messaging. However, modality preference results showed that individuals might prefer to receive public safety and emergency alerts by text message.

**Conclusions:**

Factors that influence proactive response towards public safety messaging might differ from emergency alerts. This pilot study has yielded results from a major mass-gathering event that can be used to understand errors that arise during public health and emergency preparedness and strengthen future disaster planning and research.

## Introduction

The United States’ history of high-profile disasters has changed the way local, state and federal governments and agencies approach disaster preparedness and response. Post 9/11 has created heightened importance for safety during mass-gatherings events occurring throughout the country. In preparation for mass gatherings, a Unified Command Center can be established to prepare for safety incidents and promote effective multi-agency coordination. Command centers assume the responsibility of operations, logistics, planning and information dissemination prior to and during mass gatherings [1].

The Super Bowl is one of the biggest annual sporting events that averages about 70,000 attendees per year, in addition to the residents living in the host city [2]. The magnitude of this event and widespread agency participation requires Super Bowl preparedness efforts to adopt Unified Command Center structure. Super Bowl LVI occurred in Los Angeles, California on February 13^th^, 2022. Under the Unified Command Center was the Emergency Operations Center (EOC), Joint Operations Center (JOC) and Joint Information Center (JIC). The EOC was the centralized location for incident management, while the JOC handled operations and executed command orders. The JIC was a multi-agency operations center where agency Public Information Officers collaborated, coordinated, and disseminated timely and consistent critical information to the public related to Super Bowl events.

The JIC established four tiers of notifications: public safety notifications, jurisdictional alert and warning, cross-jurisdictional alert and warning, and county-wide alerts. Public safety messaging has the potential to save lives by identifying and delivering critical information to fellow agencies and communities at risk [3]. Dissemination of timely and accurate information can keep individuals aware of ongoing events, instruct groups of protective actions, and optimize the overall safety within communities [4]. An estimated 97% of Americans own a cellphone, with almost 85% of that group owning a smartphone [5]. Therefore, the advancement and increased ownership of technology has allowed organizations and governments to improve public safety outreach through SMS messaging and social media [4, 6, 7].

The Super Bowl LVI JIC prioritized public safety messaging throughout their operational periods. They specifically partnered with Alert SouthBay, a non-profit organization that specializes in emergency alert and warning notifications through a cross-jurisdictional notification system. While partnering with Alert SouthBay, the JIC was able to create a notification platform to send Super Bowl related public safety alerts through SMS messaging.

The purpose of this pilot study is to learn from those that opted-in to the unique SMS-based notification platform deployed during Super Bowl LVI and inform future research into the effectiveness of the public safety messaging during mass-gathering events.

### Literature review

There are two theories that address how risk communication can be applied to individual decision making: Elaboration Likelihood Model of Persuasion (ELM) and Protective Motivation Theory (PMT). The ELM theory explains how an individual’s cognitive information processing affects decision making and behavior change. This model describes two cognitive routes, central and peripheral, that influence an individual’s behavior. The central route focuses on an individual’s objective analysis of incoming information, specifically its accuracy and relevancy. On the other hand, peripheral routes are inferences that individuals make regarding incoming information based on various cues and perceptions [8, 9]. Those processing primarily with central routes are more predictable in persuasion because they use objective and logical reasoning, compared to those operating primarily with peripheral routes [10]. However, these two routes operate on a continuum as one processes information and can assist in evaluating messaging factors that influence behavior change [8].

The PMT defines protective motivation as motivation that “arouses, sustains and directs” safety-related protective behaviors. The theory suggests that the two cognitive processes affiliated with protective motivation are threat appraisal and coping appraisal. The main elements within these respective processes are perceived threat and perceived efficacy, or perceived ability to perform a protective behavior [11].

ELM was originally used to analyze decision making with regards to politics and voter behavior [12]. However, in more recent research, ELM theory and PMT have both been adapted and used to understand individuals’ health decisions and improve public health strategies [13–15]. There have been few applications of these theories towards disaster management, specifically with respect to how individuals respond to SMS-based messaging. Yoo et al. recently proposed a framework using these two theories to assess proactive behavior with regards to SMS-based emergency alerts [9]. However, the effect of SMS-based public safety messaging on the public at-risk is unclear [16]. There is currently a gap in the literature, as there is no developed theoretical model to help us understand how public safety messaging can induce proactive safety decision-making. Instead, most of the literature in this field focuses on emergency alert warnings. While emergency alerts and public safety messaging are similar, the lack of an imminent event in public safety messaging could alter any existing frameworks attempting to predict behavior [17].

### Conceptual framework

**Fig 1:**
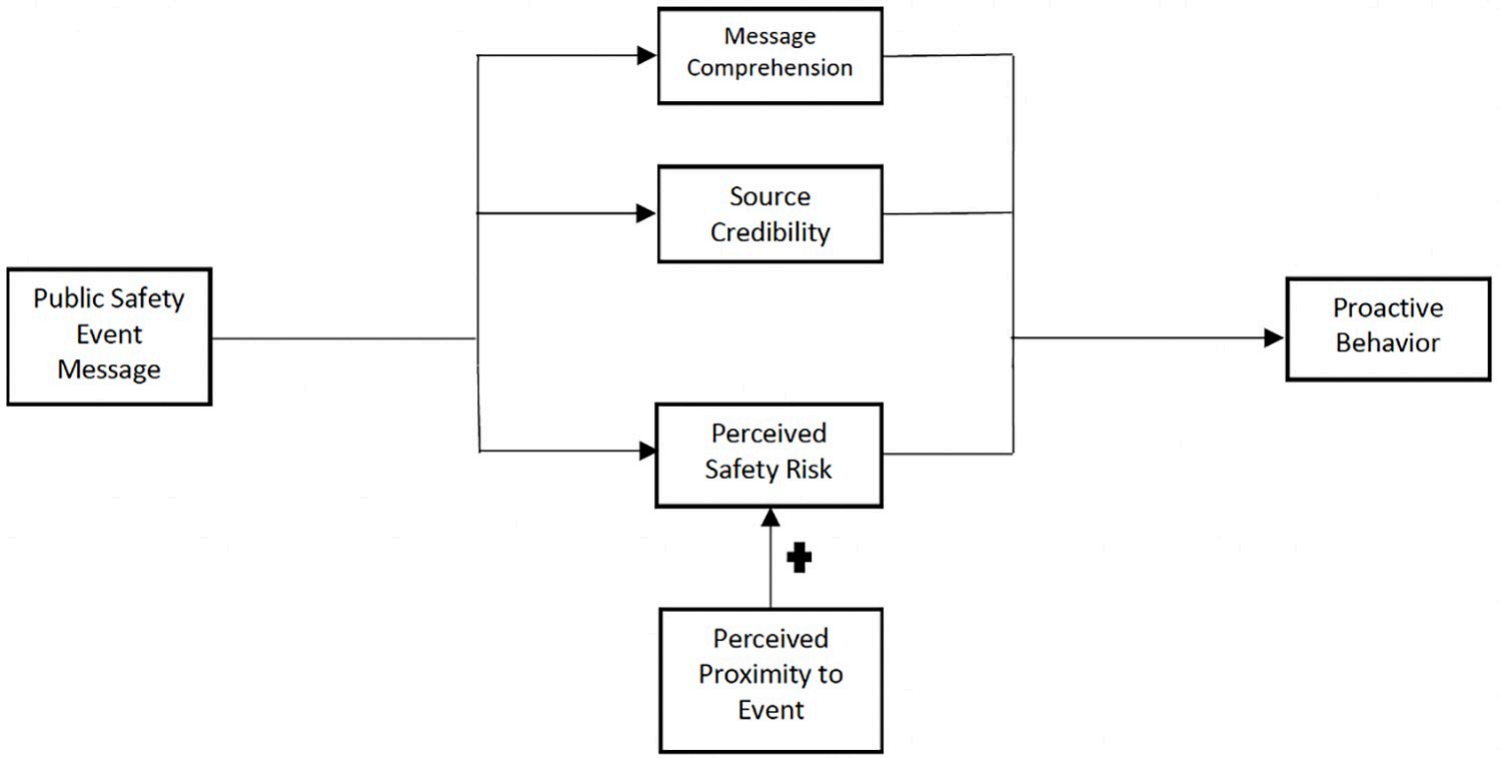
Conceptual framework.

Our pilot study sought to develop a novel conceptual framework that addresses how SMS-based public safety messaging influences proactive behavior. Our framework (Fig 1) is derived from Elaboration Likelihood Model of Persuasion (ELM), Protective Motivation Theory (PMT), and the Yoo et al. existing model that focus on emergency disaster alerts [9]. The subsections will explain the reasoning of our chosen independent variables, dependent variable, and hypothesis development. Overall, our framework will be used to study the factors that influence the relationship between public safety event messaging and proactive behavior.

According to ELM literature, an individual will use central and peripheral processing to be persuaded. Central processing leads to more objective analysis of an incoming stimulus, while peripheral processing utilizes subjective information for quick decision making. Message comprehension is the moderating factor to enter a central processing route. Source credibility, the degree to which an individual believes a person/organization that sends them information, is a significant component of peripheral processing. We hypothesize that message comprehension and source credibility can independently have a positive correlation on proactive safety behaviors.

**H1: Message comprehension positively influences proactive behavior.**

**H2: Source credibility positively influences proactive behavior**

Based on the literature, threat appraisal is one of the two processing mechanisms for the PMT model. Threat appraisal is also referred to as perceived risk [18, 19]. Those that perceive a high-risk event is personally imminent are more likely to exhibit behavior change, if they believe they have the capability to keep themselves safe [20, 21]. Given that our study’s public safety messaging was sent for general safety awareness rather than an impending disastrous event that requires immediate action, we focused on measuring perceived risk as personal relevance for safety.

**H3: Perceived risk positively influences proactive behavior**

We suggest that a moderating factor to perceived risk is proximity to an event. Identifying one’s location in relation to an upcoming event has the potential to influence behavior. Studies focusing on natural disasters have found a mainly positive relationship between hazard proximity and perceived risk [22–24]. Given that the Super Bowl was a pre-determined and well-advertised mass gathering, we believe that individuals would have no challenges approximating their location with respect to the Super Bowl events. Therefore, we postulate that when proximity to a mass gathering event is close, perceived risk will increase leading to proactive behavior.

**H4: Proximity to an event positively moderates the relationship between perceived risk and proactive behavior.**

Proactive behavior, or coping, is a commonly used as the dependent variable of study for evaluating effectiveness of emergency alerts. Specifically, task-focused coping or the completion of tasks related to enhancing safety, is used to assess adaptive behavior change [9, 20]. Given that our public safety messaging was for awareness, rather than instruction, our dependent variable was measured by evaluating whether one read the message and/or sought out credible sources for further information.

## Methods

### Target population and recruitment

The Alert SouthBay and JIC notification platform was established through Everbridge, an all-channel, critical communication system used to communicate with all individuals located in a specific jurisdiction through SMS-based messaging [25]. This platform was widely marketed to through sign-boards off exits close to SoFi stadium, agency social media posts, and hotel flyers to locals and visitors alike to reduce selection bias. Marketing material included a short description of services and an opt-in keyword “INGSB” that an individual could text to sign up for the notification system. Once individuals signed up for the Super Bowl notification platform, the JIC could send direct SMS messages to this group with relevant public safety information.

### Public safety message

One public safety message was sent the morning of Super Bowl LVI. The text message stated that Super Bowl LVI game day information was available for all participants and prompted them to click a link for a public safety message. This public safety message highlighted current traffic, parking, and highway concerns, while also addressing the heat advisory issued for the day. The message concluded by listing current social media platforms that individuals could use to get up-to-date information for any public health information throughout the day.

### Instrument development

Table 1 illustrates the survey design instrument disseminated to measure our variables. These statements were presented with respect to the two sets of messages that participants received: the initial text message notification and the public safety message provided as a link in the text message. For example, a picture of the initial text message was shown, and participants responded to the statement “I understood this message clearly”. Later, the participants were showed the public safety message and responded to the same statement “I understood this message clearly”. Every statement was accompanied by a 5-Point Likert Scale from strongly disagree (0) to strongly agree (5).

**Table 1:** Survey measurement items. 5-Point Likert Scale.

Variable with Corresponding Survey Statement
Message Comprehension
Statement: *I understood this message*
Statement: *This message was written clearly*
Source Credibility
Statement: *I trusted this message*
Perceived Risk
Statement: *I took this message seriously*
Statement: *I believed this message was related to my safety*
Proactive Behavior
Statement: *I read this message*
Statement: *I clicked the link(s) provided for further information*

### Data collection and analysis

Within 11 days of the Super Bowl, a text message was sent to all participants that included a link to a Qualtrics^xm^ web-based survey to assess their responses to the notification platform and public safety message that was sent during the Super Bowl. A reminder text message was sent a couple of days after the original survey link was disseminated to boost survey participation. To improve recollection of events, the survey included the date, time and pictures of the exact messages that were sent. There was no compensation awarded for participation.

The survey was only disseminated by the same SMS-based messaging system that was used to disseminate Super Bowl LVI notifications, but was made accessible for mobile devices, tablets and laptops. The survey was also translated into Spanish with the help of the Public Information Officers (PIOs) within the JIC that were native-Spanish speakers. Individuals were given 48 hours to complete the survey before their responses were recorded. Partial responses that reported a 63% or higher completion rate were included within analysis. This ensured recipients answered enough of the questions in the survey for appropriate analysis.

Data was analyzed anonymously using STATA Statistical Software. The need for informed consent was waived by the ethics committee, as there was no identifiable information collected. This survey design study was approved by University of Southern California’s Institutional Review Board (Approval #APP-22-01755).

## Results

### Demographic and safety characteristics

There was a total of 582 individuals signed up for this Super Bowl notification platform. A total of 57 participants completed the survey. Of those, three participants completed less than 63% of the survey and were excluded from analysis. The total sample size is 54 participants (*N = 54)*. The majority of respondents were female (65.5%), between 25 to 49 years of age (54.5%), and attained at least a Bachelor’s degree (67.3%). Over a third of respondents were older than 50 years of age. Most of the respondents were African American (32.7%), Hispanic (27.3%), or Caucasian (23.6%) (Table 2).

**Table 2:** Demographic descriptive statistics (N = 54).

Variable	N (%)
*Sex*	
Male	17 (30.9%)
Female	36 (65.5%)
Prefer not to say	1 (1.8%)
*Age (Years)*	
19–24	3 (5.5%)
25–49	30 (54.5%)
60–65	13 (23.6%)
65+	8 (14.5%)
*Race/Ethnicity (Select all that Apply)*	
American Indian or Alaskan Native	1 (1.8%)
American Indian & Black	1 (1.8%)
Asian & White/Caucasian	1 (1.8%)
African American or Black	18 (32.7%)
Hispanic	15 (27.3%)
Native American	1 (1.8%)
Non-Hispanic White	13 (23.6%)
Other	4 (7.3%)
*Education*	
High school or GED	7 (12.7%)
Associate’s Degree	10 (18.2%)
Bachelor’s Degree	20 (36.4%)
Graduate Degree	17 (30.9%)

General safety questions were asked to understand the group that opted in for the notification platform in relation to Super Bowl LVI. A majority of respondents are residents of Los Angeles (72.7%) compared to Super Bowl visitors (25.5%). Most were in very close proximity to Super Bowl events (67.3%). A majority of participants stated that text message was the best way to reach them in case of an emergency (83.6%) (Table 3).

**Table 3:** Safety characteristics ^a^.

Variable	N (%)
*Do you live in Los Angeles*?	
Yes	40 (74%)
No	14 (26%)
*How close were you to the Super Bowl*?	
Not Close at All	6 (11.1%)
Somewhat Close	11 (20%)
Very Close	37 (68.6%)
*What is the best way to reach you during an emergency*?	
Text Message	46 (83.6%)
Email	1 (1.8%)
Social Media	2 (3.6%)

^a^: Total percent might not add up to 100 percent due to missing values

### Correlation of variables

There were two questions each used to measure message comprehension, perceived risk, and proactive behavior. Spearman’s correlation was used to determine whether these measurements were correlated to each other. Message comprehension and perceived risk measurements were highly correlated (p < 0.001). Further analysis used the measurements “I understood this message” and “I took this message seriously” for each respective variable. Proactive behavior measurements were not correlated with each other and further analysis created a composite score from each of the measurements.

### Association of independent variables to proactive behavior

Message comprehension, source credibility and perceived safety risk were analyzed through chi-squared tests (Fisher’s Exact) to find their association with respect to proactive behavior. Due to small sample sizes, the 5-Point Likert Scale was reduced to a 3-Point Likert scale of disagree, neutral and agree.

None of the independent variables for text message or public safety message were found to have a significant association with proactive behavior. Additionally, there was no significant effect modification between perceived safety risk and proactive behavior by perceived proximity to Super Bowl Events (Table 4).

**Table 4:** Correlation of messaging factors on proactive behavior.

*Message Factor*	*Fisher’s Exact Value*	*P-Value (2 Sided)*
Message Comprehension		
*Text Message*	9.031	0.062
*Public Safety Message*	5.288	0.245
Source Credibility		
*Text Message*	4.661	0.411
*Public Safety Message*	6.682	0.09
Perceived Safety Risk		
*Text Message*	7.342	0.095
*Public Safety Message*	6.841	0.086
Proximity to Event (Effect Modification)		
*Text Message*	7.342	0.095
*Public Safety Message*	6.841	0.086

## Discussion

Most studies in disaster communications literature focus on messaging during emergencies with little research extending into public safety messaging. Our pilot study intended to modify the most current framework, proposed by Yoo et al., to inform future research regarding communication factors that influence response to public safety messaging and overall emergency preparedness as it relates to mass gatherings. Super Bowl LVI provided a unique platform to deploy this pilot framework. The JIC created a Super Bowl specific SMS-based messaging system to send public safety alerts to all individuals that opted-in during the week-long activation period. Given that there were no emergencies, there was one public safety message sent on the morning of the Super Bowl. Our pilot then used this framework to gather data from the responses to this public safety message.

The ELM theory and PMT propose that the cognitive processes of message comprehension, source credibility and perceived risk can influence positive and protective behavior decisions. Research has proven that these factors were found to be significant predictors of proactive behavior when assessing emergency alert messaging systems [6, 9]. However, this pilot study found no association between these independent variables and proactive behavior. This calls into question whether the same communication factors are truly influential for public safety messaging during mass gathering events. While this pilot does not allow for generalizability, the results do provide critical information to inform future research trying to uncover whether the same holds for public safety messaging.

In times of emergency, individuals’ priorities are influenced by a variety of cues to make quick, actionable decisions [17, 26, 27]. However, given that public safety messages are sent to prepare for future emergencies, we could postulate that respondents have more time to engage in simple proactive behaviors. Additionally, it is possible that respondents rely on certain factors differently when they are not in imminent danger.

The demographics of survey respondents revealed a widely diverse group. Racially, the respondents were similar to the make-up of Inglewood and its surrounding Super Bowl locations. In terms of age, most respondents were between 25 and 65 years old. Interestingly, the smallest group of respondents were under 24 years old, the group we consider to be the highest technological users [5]. Although this is not reflective of who opted-in to the notification platform, it still raises a question as to why there was a difference in survey participation. In general, mobile surveys with appropriate design and SMS invitation have been found to an effective way of gathering survey data from a vast population group [28, 29]. However, further consideration should be given to whether survey dissemination solely through text message was responsible for the low response rate in certain groups.

Past research has proven that text message and mobile apps are a practical and effective tool for public health and emergency communications [30–32]. Specifically, mass gathering events create high level ambient noise that makes other forms of verbal communication challenging [33]. Our results revealed that almost 84% of respondents favored text message as an emergency communication modality. This might reflect an adapted change in preferences, as the aging population has increased their use of text messaging [34, 35], even in emergency situations. More specifically, while social media has been found to spread information quickly, text message remains most effective as a one-on-one, non-confounded messaging tool [36]. Our survey was only disseminated by text message, which could have led to bias in respondents choosing text message as a preferred modality. However, further research should explore modality preferences in the context of public safety message dissemination, given that text message has been found to be preferred in other public health contexts previously.

### Limitations and recommendations

Our pilot study’s major limitations were the survey response rate and lack of generalizability. The results of this study might reflect the characteristics of a very limited group compared to all of those that opted-in for the Super Bowl notification platform. Dissemination of the survey solely by text message could have impacted the number, type and quality of the responses that were received. Future research should employ alternative survey instruments and techniques to reach a wider audience and reduce biases in responses. Further research is encouraged to garner increased research participation and generalizable results.

Secondly, our survey data depended on respondents remembering how they reacted towards the public safety message sent 11 days prior. Our pilot provided the actual public safety messaging in the survey as a strategy to aid recall [37]. However, responses could have still been subjected to recall bias, which could have affected overall responses. Future research should expedite survey dissemination to reduce this phenomenon.

Although this study was developed based on validated theoretical frameworks of ELM and PMT, the survey tool itself is not validated, which further limits any generalizability. Further studies should seek to validate a survey tool that can effectively assess individuals’ responses to public safety messaging.

## Conclusion

Despite these limitations, this study contributes to the public health literature at large. Little research has been done applying communication theory to public safety alerts, especially during mass-gathering events. This unique pilot study modified and deployed a novel framework during Super Bowl LVI to understand how and why individuals responded to public safety messaging. The results call into question whether public safety alerts are influenced by similar factors that alter receptiveness to emergency alerts. This implicates future research seeking to understand how public safety messaging can be more effective in encouraging individuals to make proactive, protective decisions. This is especially important during mass gathering events, where a large group of people are threatened by a numerous amount of health hazards [38]. This study should be treated as a pilot study that highlights potential errors in the receptiveness of public safety messaging which should be taken into consideration to strengthen future disaster planning research and outcomes for mass-gathering events.
